# Peroxisome proliferator-activated receptorα/γ agonist pioglitazone for rescuing relapsed or refractory neoplasias by unlocking phenotypic plasticity

**DOI:** 10.3389/fonc.2023.1289222

**Published:** 2024-01-11

**Authors:** Dennis Christoph Harrer, Florian Lüke, Tobias Pukrop, Lina Ghibelli, Christopher Gerner, Albrecht Reichle, Daniel Heudobler

**Affiliations:** ^1^ Department of Internal Medicine III, Hematology and Oncology, University Hospital Regensburg, Regensburg, Germany; ^2^ Division of Personalized Tumor Therapy, Fraunhofer Institute for Toxicology and Experimental Medicine, Regensburg, Germany; ^3^ Bavarian Cancer Research Center (BZKF), University Hospital Regensburg, Regensburg, Germany; ^4^ Department of Biology, University of Rome Tor Vergata, Rome, Italy; ^5^ Department of Analytical Chemistry, Faculty of Chemistry, University of Vienna, Vienna, Austria

**Keywords:** pioglitazone, interferon-α, dexamethasone, all-trans retinoic acid, tumor tissue editing, anakoinosis, transcriptional modulation, phenotypic plasticity

## Abstract

A series of seven clinical trials on relapsed or refractory (r/r) metastatic neoplasias followed the question: Are networks of ligand-receptor cross-talks that support tumor-specific cancer hallmarks, druggable with tumor tissue editing approaches therapeutically exploiting tumor plasticity? Differential recombinations of pioglitazone, a dual peroxisome-proliferator activated receptor**α/γ** (PPARα/γ) agonist, with transcriptional modulators, i.e., all-trans retinoic acid, interferon-α, or dexamethasone plus metronomic low-dose chemotherapy (MCT) or epigenetic modeling with azacitidine plus/minus cyclooxygenase-2 inhibition initiated tumor-specific reprogramming of cancer hallmarks, as exemplified by inflammation control in r/r melanoma, renal clear cell carcinoma (RCCC), Hodgkin’s lymphoma (HL) and multisystem Langerhans cell histiocytosis (mLCH) or differentiation induction in non-promyelocytic acute myeloid leukemia (non-PML AML). Pioglitazone, integrated in differentially designed editing schedules, facilitated induction of tumor cell death as indicated by complete remission (CR) in r/r non-PML AML, continuous CR in r/r RCCC, mLCH, and in HL by addition of everolimus, or long-term disease control in melanoma by efficaciously controlling metastasis, post-therapy cancer repopulation and acquired cell-resistance and genetic/molecular-genetic tumor cell heterogeneity (M-CRAC). PPARα/γ agonists provided tumor-type agnostic biomodulatory efficacy across different histologic neoplasias. Tissue editing techniques disclose that wide-ranging functions of PPARα/γ agonists may be on-topic focused for differentially unlocking tumor phenotypes. Low-dose MCT facilitates targeted reprogramming of cancer hallmarks with transcriptional modulators, induction of tumor cell death, M-CRAC control and editing of non-oncogene addiction. Thus, pioglitazone, integrated in tumor tissue editing protocols, is an important biomodulatory drug for addressing urgent therapeutic problems, such as M-CRAC in relapsed or refractory tumor disease.

## Introduction

Pioglitazone is a thiazolidine-2, 4-dione compound and an approved dual peroxisome proliferator-activated receptor (PPAR)α/γ agonist for the treatment of insulin resistance (1, 2). Besides this limited indication, more and more pre-clinical data reveal a broad, multileveled activity profile in cancer tissue, modulating cancer-associated inflammation, immune response, sustained proliferative signaling, cancer metabolism, angiogenesis, i.e., tissue functions that are described by the hallmarks of cancer (3, 4).

Promising experimental findings on the anti-tumor activity of pioglitazone face the difficulty of missing monoactivity in metastatic tumor disease, despite of the very low mutation rate of PPARα/γ in human tumor cells (5–7). Additionally, the use of glitazones in clinical settings is pejorative because dual PPARα/γ agonists may induce histologically different tumors in rodents (8). However, the discussion about pioglitazone and bladder cancer induction in patients with type II diabetes is still not based on sufficient evidence (9). Research for novel, more active PPARα/PPARγ/PPARα/γ agonists did not bring more agonists to clinical approval (10).

Two rationales worked hand in hand to successfully integrate pioglitazone in novel therapy designs for relapsed/refractory (r/r) metastatic neoplasias. Firstly, a general therapeutic problem is pending for solution. Maximized apoptosis induction with maximum tolerable doses of pulsed therapies for rescuing r/r tumor states may inevitably re-establish in most neoplasias cancer promoting hallmarks, metastasis, post-therapy cancer repopulation and acquired cell-resistance and genetic/molecular-genetic tumor cell heterogeneity (M-CRAC) and limits initially induced tumor responses (11, 12). Thus, approved pulsed therapy approaches- irrespectively of their composition – are extremely challenged to induce continuous complete remission (cCR) and, therefore, demand novel therapy techniques to overcome M-CRAC (13).

Secondly, novel therapy techniques resolving disease traits of M-CRAC must unlock the tumor phenotype by reprogramming hallmarks of cancer, just those included in M-CRAC, that means, multiple tumor cell compartments and their communication profiles must be targeted, as hypothesized, by the concerted use of transcriptional modulators, including pioglitazone (14–19).

As clinically shown, unlocking the tumor’s plasticity with therapy approaches editing the tumor’s growth-promoting phenotype in a therapeutically relevant way, i.e., tumor tissue editing, facilitates the successful clinical integration of combined transcriptional modulation with pioglitazone (20–24). Tumor tissue editing techniques concertedly reprogram hallmarks of cancer for establishing biologic hallmarks that may control tumor growth, and even induce tumor cell death (11).

Tissue editing techniques include synergistic combinations of bioactive drugs, such as metronomic low-dose chemotherapies, transcriptional modulators, i.e., pioglitazone, interferon-α, dexamethasone or all-trans retinoic acid (ATRA) and cyclooxygenase-2 (COX-2) inhibitors, and may induce long-term tumor control, objective response, even continuous complete remission (cCR) in r/r neoplasias (21–23, 25–28). On the top, edited stress reponse pathways, referred to as non-oncogene addiction in tumor tissues, provide a novel, repurposed activity profile for approved targeted therapies (20, 29).

Inflammation control, reestablishing immunosurveillance, metabolic reprogramming, enhancing tumor growth suppression, differentiation induction and consecutively M-CRAC control are now the contributions of pioglitazone within editing approaches, as shown in a large series of clinical trials (21–23, 30–32).

The present review categorizes the reprogramming activity of pioglitazone in recombination with additional transcriptional modulators, also that of metronomic low-dose chemotherapy, by analyzing trials on tumor tissue editing, designed for promoting inflammation control or differentiation induction in r/r metastatic cancer or r/r non-promyelocytic acute myelocytic leukemia (non-PML AML).

## Tissue editing as prerequisite for integrating pioglitazone into the repertoire of systemic tumor therapies

The novel treatment concept ‘tumor tissue editing’ adopts to the use of tissue editing techniques for correcting epigenetic or genetic aberrations in tumor tissues (20). Tumor tissue editing methods aim at therapeutically exploiting tumor phenotypes by reprogramming hallmarks of cancer (28).

Tumor tissue editing is defined by therapy-guided targeted evolution of tumor tissues for establishing biologic functions in tumor tissues facilitating tumor control or initiating complete remission in relapsed or refractory tumor disease.

**Figure 1:**
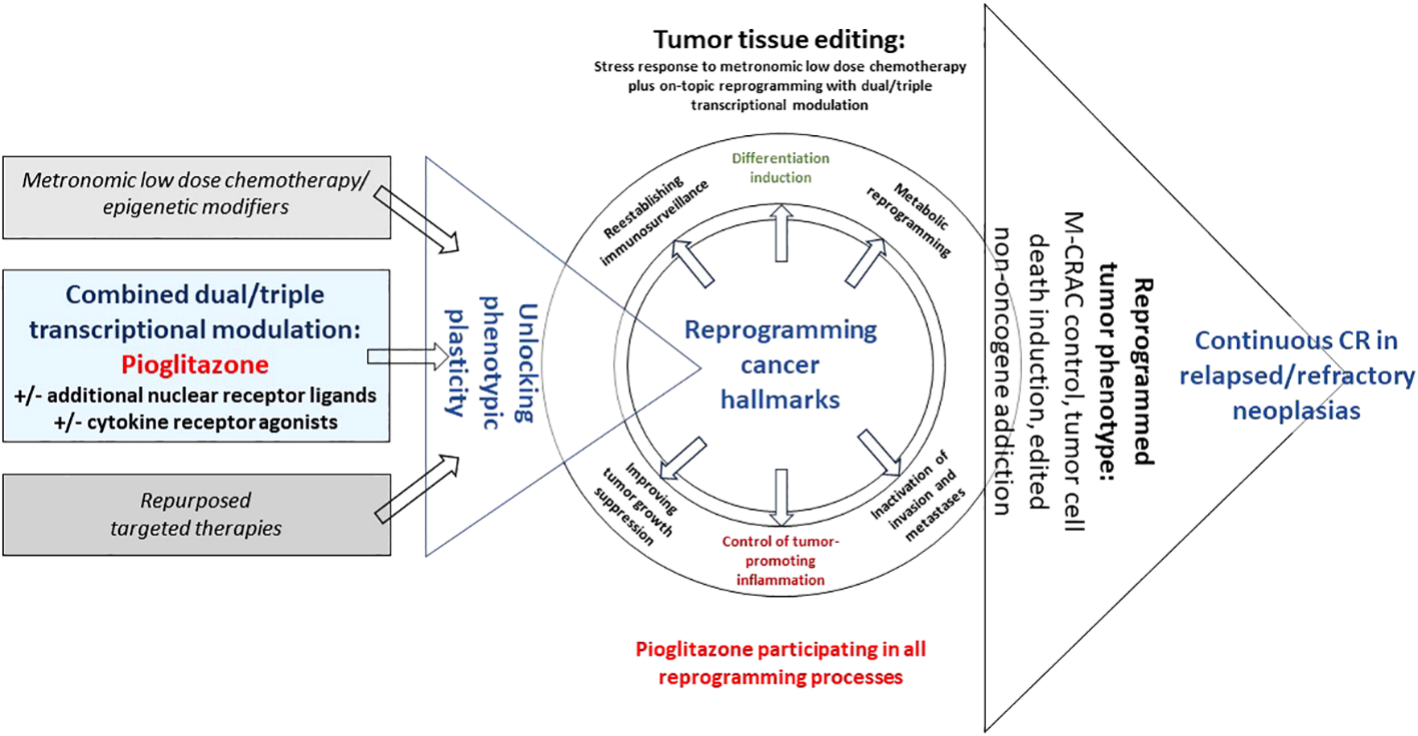
Unlocking tumor phenotype with editing schedules including pioglitazone. Editing with pioglitazone may reprogram hallmarks of cancer via tumor-specific ligand-receptor networks, thereby, inducing tumor cell death, M-CRAC control and edited non-oncogene addiction in relapsed/refractory metastatic tumor disease or hematologic neoplasias.

Tumor tissue editing techniques are supported by bioactive drugs with no or poor monoactivity that are therapeutically involving tumor cells but as well, the whole repertoire of stroma cells, i.e., hematopoietic cells, such as myeloid-derived suppressive cells, tumor-associated macrophages (TAMs) and T-cells, mesenchymal cells, cancer-associated fibroblasts (CAFs), lipocytes, and endothelial cells (26, 33). The target of metronomic low dose chemotherapy plus transcriptional modulators is the tumor tissue’s phenotypic plasticity to be therapeutically exploited (**Figure 1**).

Backbone of the editing concept is pioglitazone and metronomic low dose chemotherapy. The addition of selected transcriptional modulators, such as ATRA, interferon-a or dexamethasone contributes to differentiation induction in r/r non-PML AML, to inflammation control in r/r Hodgkin’s lymphoma, Langerhans cell histiocytosis and RCCC, and clinically documented impressive immune response in CRPC (22, 23, 34, 35). All these biologic surrogates, inflammation control, differentiation induction and immune response may be associated with objective tumor response, CR or cCR in r/r neoplasias (**Table 1**).

**Table 1:** Pioglitazone contributing to M-CRAC control or resolution in carcinomas, sarcomas and hematologic diseases with tissue editing approaches.

Transcriptional regulation	MCT, targeted therapy	relapsed/refractory neoplasia	Reprogramming cancer hallmarks	Best response	Common therapies for relapsed/ refractory disease
**Randomized** rofecoxib plus/minus pioglitazone	MCT (trial 1)	**Metastatic melanoma**	Inflammation control	PR	Immune checkpoint inhibitors, targeted therapies, chemotherapy
Pioglitazone, etoricoxib	MCT+ temsirolimus	**Metastatic uveal melanoma**	Edited non-oncogene addiction	Long-term SD	Immune checkpoint inhibitors, targeted therapies, chemotherapy
Pioglitazone, rofecoxib	MCT	Cholangiocellular carcinoma	n.d.	**cCR**	Targeted therapies, chemotherapy
Pioglitazone, rofecoxib	MCT	Hepatocellular carcinoma	Inflammation control	PR	Immune checkpoint inhibitor, targeted therapies, chemotherapy
**Randomized** rofecoxib plus/minus pioglitazone	MCT	Gastric cancer	n.d.	PR	Chemotherapy, immune checkpoint inhibitors, trastuzumab deruxtecan, anti-angiogenetic therapy
Pioglitazone, rofecoxib	MCT	High-grade gliomas	n.d.	SD	Chemotherapy, radiotherapy
Pioglitazone, rofecoxib	MCT	Angiosarcoma	n.d.	**cCR**	Chemotherapy
Pioglitazone, etoricoxib **Randomized** vs. nivolumab	MCT, clarithromycin	r/r Non-small cell lung cancer	Immune modulation	PR, suggested improved activity of immune checkpoint inhibitors in further progression	Immune checkpoint inhibitors, targeted therapies
Pioglitazone, dexamethasone, etoricoxib	MCT (trial 1) + Imatinib (trial 2)	Castration-refractory prostate cancer	After therapy discontinuation long-term stable disease Reconstitution of hormone sensitivity	PR	Chemotherapy, PARP inhibitor
Pioglitazone, dexamethasone, etoricoxib	MCT, everolimus	**Hodgkin‘s lymphoma**	Inflammation control **Edited non-oncogene addiction** (mTOR)	**cCR** **cCR** following consecutive allogeneic HSCT in CR	Immune checkpoint inhibitor, chemotherapy, brentuximab, autologous PBSCT
Pioglitazone, dexamethasone, etoricoxib	MCT	**Multisystem Langerhans cell histiocytosis**	Inflammation control	**cCR**	Chemotherapy
Pioglitazone, etoricoxib plus interferon-α	MCT (trial 1) MCT (trial 2)	**Renal clear cell carcinoma**	No inflammation control Inflammation control	SD **cCR**	Immune checkpoint inhibitors, tyrosine kinase inhibitors, mTOR inhibitor
Pioglitazone, dexamethasone	MCT, lenalidomide	Multiple myeloma	after discontinuation long-term stable disease, **resolution of IMiD resistance**	PR	CAR-T cells, bispecific antibodies
Pioglitazone, all-trans retinoic acid	Azacytidine	**Non-promyelocytic acute myelocytic leukemia**	**Differentiation induction**	**CR**, molecular-genetic, hematologic **cCR** following consecutive allogeneic HSCT in CR	Chemotherapy, targeted therapies

Each treatment schedule consisted of metronomic chemotherapy and dual/triple transcriptional modulation including pioglitazone. CR and cCR indicates resolution of M-CRAC in relapsed/refractory (r/r) disease. Possible common palliative ‘standard’ rescue therapies for respective r/r metastatic tumor diseases are listed. MCT, metronomic low dose chemotherapy (capecitabine, treosulfan, trofosfamide), SD, stable disease, PR, partial response, CR, complete remission, cCR, continuous CR, HSCT, hematopoietic stem cell transplantation, mTOR, mammalian target of rapamycin, tumor diseases in bold, discussed in more detail.

Currently, the concerted activity profile of the biomodulatory drug combinations cannot be pinned down to single pathways or to the contribution of single cell types of the tumor tissue. The necessity to use drug combinations including pioglitazone has been exemplarily pre-clinically and clinically tested in case of non-PML AML or clinically with consecutively performed trials with identical inclusion criteria (r/r RCCC) or randomized phase II trials (34, 36–39).

Thus, the clinical results rely on a concerted activity profile of drugs without monoactivity in the respective histologic tumor type. The suggested mechanism of action is a communicative reprogramming of tumor phenotypes. That is a novel approach for controlling r/r neoplasias and M-CRAC development.

Although, the activity of the single drugs used in editing approaches is basically pharmacologically well-defined, their interaction with additional transcriptional modulators is mostly poorly understood, especially on the background that metronomic low dose chemotherapy is essential to establish the striking activity of the combinations of transcriptional modulators. The drug combinations only, lead to clinical effects, such as CR or cCR in r/r neoplasias (**Table 2**).

**Table 2:** Editing schedules for differentiation induction in relapsed/refractory (r/r) acute myelocytic leukemia and inflammation control in r/r renal clear cell carcinoma, r/r Hodgkin’s lymphoma and r/r metastatic melanoma are exemplarily shown.

Differential tumor tissue editing approaches for differentiation induction or inflammation control				
	Tissue editing schedule for relapsed or refractory non-promyelocytic acute myelocytic leukemia, one trial: differentiation induction			
ATRA 45 mg/m²/day	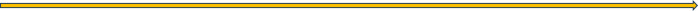			
Azacitidine 75 mg/day	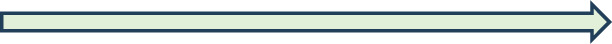			
Pioglitazone 45 mg/day	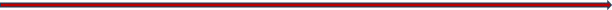			
	Tissue editing schedule for relapsed or refractory renal clear cell carcinoma (RCCC), two trials; inflammation control			
Pioglitazone 60 mg/day	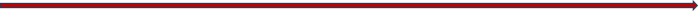			
Capecitabine 1g BID x2 weeks	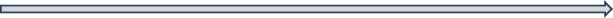			
+/- **interferon-α** 4.5 MU s.c. three times a week	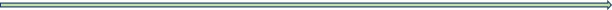			
Rofecoxib 25 mg/day	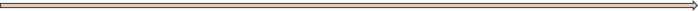			
	Tissue editing schedule for relapsed or refractory metastatic melanoma, two trials: inflammation control, edited non-oncogene addiction			
+/-Pioglitazone 60 mg/day	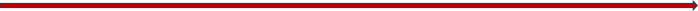			
Trofosfamide 25 mg thrice/day	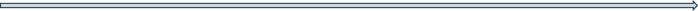			
+/- **Temsirolimus** 25 mg/ week	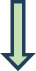	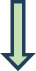	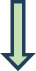	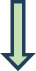
Rofecoxib 25 mg/day	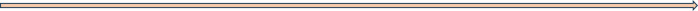			
	Tissue editing schedule for relapsed or refractory Hodgkin‘s lymphoma, one trial: inflammation control, edited non-oncogene addiction			
Pioglitazone 45 mg/day	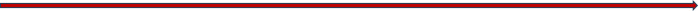			
Treosulfan 250 mg twice/day	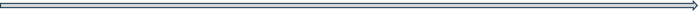			
**Everolimus** 15 mg/day	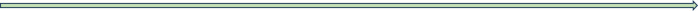			
Etoricoxib 60 mg/day	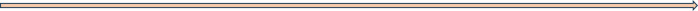			
Dexamethasone 0.5 mg/day	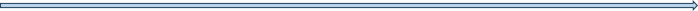			
day	7	14	21	28

In Hodgkin’s lymphoma and uveal melanoma, editing of non-oncogene addiction is possible.

‘Normalizing’ tumor-promoting pro-inflammatory signals from quite different sources or redirecting sustained proliferation by differentiation induction are activity profiles of pioglitazone *in vitro* and in animal models (40–43). Unsurprisingly, in heterogeneous and dynamically evolving tumor systems, the activities of drugs that are not directly targeting oncogenic events, tumor cell specific targets or non-oncogene addiction, but predominantly participate in the realignment of dysbalanced homeostatic processes among cancer hallmarks, show poor or clinically irrelevant monoactivity, like pioglitazone and other agonistically active transcriptional modulators (26).

Pioglitazone plus/minus recombined transcriptional modulators contributes to phenotype plasticity of metastatic r/r neoplasias by facilitating phenotype switches, associated with inflammation control or differentiation, under tumor tissue conditions established by metronomic low-dose chemotherapy (**Table 2**). Immediate therapeutically induced phenotype switches may be functionally or morphologically comprehensible, but are not based on genetic changes as those frequently discussed in terms of ‘phenotype changes’ (36, 44).

Tumor tissue editing as systems-biologic therapy approach targets tumor-specific networks of ligand-receptor crosstalks, i.e., pro-anakoinotic activity, and reprograms complex homeostatic dysbalances of cancer hallmarks in a therapeutically meaningful way (**Figure 1**) (17, 22, 28, 45).

Therapeutically reprogramming sustained tumor cell proliferation by control of tumor-linked inflammation, by improving immunosurveillance, correcting tumor tissue’s metabolic processes, reestablishing tumor suppression, inducing differentiation, proved to be systematically druggable approaches by the introduction of tumor tissue editing techniques that may achieve tumor cell death, attenuation, resolution or bypassing of M-CRAC and edited non-oncogene addiction in r/r neoplasias of quite different histologic origin (**Table 3**; **Figure 1**) (3, 22, 28).

**Table 3:** Tumor tissue editing trials for different relapsed/refractory tumors and hematologic neoplasias are categorized according to metronomic low-dose chemotherapy/azacitidine, transcriptional modulation with pioglitazone and respective recombinations with further transcriptional modulators aiming at inflammation control, differentiation induction, and at editing non-oncogene addiction, here, *mTOR addiction in r/r Hodgkin’s lymphoma and uveal melanoma.

Tumor tissue editing schedules including dual/triple transcriptional modulation with pioglitazone: Best response and edited non-oncogene addiction								
		Dual/triple transcriptional modulation	Tumor histology	Best response	Targeting edited non-oncogene addiction (mTOR inhibitor)	Tumor histology	Best response	Literature
**Inflammation control**	Metronomic low dose chemotherapy plus cyclooxygenase-2 inhibitor	**Without** pioglitazone ® **Plus** pioglitazone	Relapsed/refractory (r/r) **metastatic** melanoma*	**PR** **PR, PFS**↑**, OS**↑ **significant**	- **+** Temsirolimus	r/r metastatic **Melanoma, uveal** melanoma*	Uveal melanoma: **Long-term SD**	**38, 50, 80, 137**
		Pioglitazone	r/r metastatic **renal clear cell** carcinoma*	**SD**	-	-	**-**	**35, 37**
		Pioglitazone **plus** Low dose interferon-α	r/r metastatic **renal clear cell** carcinoma*	**cCR**	-	r/r multisystem **Langerhans cell histiocytosis** (rescue therapy)	**cCR**	**23, 37**
		Pioglitazone **plus** Low dose dexamethasone	r/r multisystem **Langerhans cell histiocytosis**	**cCR**	**+** Everolimus	r/r metastatic **Hodgkin‘s** Lymphoma*	**cCR**	**23**, **126** **22**, **148**
**Differentiation induction**	Low dose azacitidine	Pioglitazone **plus** All-trans retinoic acid	r/r **Non-promyelocytic** **acute myelocytic leukemia**	**Hematologic, molecular** **CR**	-	-	**-**	**34**

R, randomization; PR, partial remission, CR, complete remission, cCR, continuous complete remission, OS, overall survival, PFS, progression-free survival, SD, stable disease, mTOR inhibitor, Target of rapamycin inhibitor.

Tumor tissue editing provides considerable clinical advantages, establishes tumor cell death by targeting the tumor’s systems biology, and the homeostatic dysbalances constituted between hallmarks of cancer. Editing saves toxicity, as only regulatorily active doses must be used. The single drugs in editing schedules must not show any clinical monoactivity, such as pioglitazone (20, 21).

## Pioglitazone as important modulator of tumor tissue’s plasticity

### Description of the target

The dual nuclear transcription factor agonist pioglitazone regulates homeostatic balances maintained by all cell compartments of the tumor tissue, the tumor cells, the tumor microenvironment with immune cells, cancer-associated fibroblasts, endothelial cells, and tumor-related adipocytes (10, 46–48). The importance of PPARγ expression in tumor cells for tumor pathophysiology is determined by the expression of the PPARγ receptor which varies dependent on tumor stage and histology n (49, 50). Receptor-independent activities of pioglitazone must be considered, additionally (26).

### Activating activity of pioglitazone

In contrast to most drugs used for tumor therapy, pioglitazone has an activating activity and modulates intra- and intercellular communication lines, including Wnt signaling (43). Contradictory results on pioglitazone concerning its activity derived from experimental studies are not astonishing as the cellular context and context-dependent interpretation of signals decisively guides the activity profile of pioglitazone, also in non-oncologic disease (26, 51). The dual activity of pioglitazone decisively extends the activity profile also in non-oncologic disease, as shown by the withdrawal of rosiglitazone, a specific PPARγ agonist (3, 52, 53). PPARα activation adds a strong anti-inflammatory effect (54, 55).

The role of PPARα/γ expression in tumor cells for response to tumor tissue editing approaches remains open, especially as metronomic chemotherapy may enhance PPARγ expression in stress response to metronomic chemotherapy (56). For example, in r/r cholangiocarcinoma, r/r Hodgkin’s lymphoma (HL) with a principally weak PPARγ expression, cCR may be achieved with editing schedules including pioglitazone, in contrast, in r/r non-small cell lung cancer with commonly relative intensive expression, only partial remission (21, 22, 39, 49). In r/r metastatic melanoma significant improved PFS was observed for patients with high PPARγ expression in respective tumor probes (49, 50).

### Pioglitazone and additional transcriptional modulators

The interaction of pioglitazone with interferon-α, dexamethasone or all-trans retinoic acid has been recently described (26). Type I interferons show synergistic antiproliferative activity if combined with glitazones in pancreatic cancer cell lines (57). In the two indicated phase II trials on RCCC, starting dose of interferon-α was only 4.5 million U thrice weekly with scheduled de-escalation dependent on tolerability as compared to the approved dose of up to 18 million U thrice weekly in monotherapy for RCCC (58). ATRA and pioglitazone show synergistic activity in AML *in vitro* and *in vivo* (30, 34, 36). The glucocorticoid receptor functions in a combinatorial manner with PPARα/γ by reprogramming and integrating local and systemic responses to inflammation (59). How the immune response of glucocorticoids and pioglitazone is modified by metronomic low dose chemotherapy cannot be pinned down, yet. Metronomic chemotherapy may additionally improve immunesurveillance, as shown by the combination of immune checkpoint inhibitors in a randomized phase III trial (60).

### Synergism, ‘coalism’, anakoinosis

**Tables 2**, **3** outline the major therapy elements of tissue editing approaches including pioglitazone that have been introduced for unlocking tumor phenotypes in r/r neoplasias. How do single drugs without significant monoactivity contribute to tumor tissue editing approaches?

Drug interactions may be considered in a traditional way. Steel et al. introduced the term ‘coalism’ for drugs that are not active alone, or active in ‘cooperation’ if the combined effect is directed on a range of biologic systems (61, 62), (63). This applies for pioglitazone. In the next step, the targeted biologic systems, and their target profiles available for reprogramming hallmarks of cancer, are of pivotal interest.

Anakoinosis outlines a novel systems-therapeutic anticancer treatment paradigm, the therapeutic unlocking and exploitation of tumor specific phenotypes for controlling r/r metastatic disease by reprogramming cancer hallmarks and ‘normalizing’ dysbalanced tumor tissue homeostasis. The selected editing techniques, and on tumor site the specific patterns of pro-anakoinotically druggable communicative tissue networks, and homeostatically balancing hallmarks of cancer, are determining the qualitative outcome of pro-anakoinotic reprogramming techniques (**Table 3**).

Still insufficiently evaluated are the tumor-specific network characteristics coordinating hallmarks of cancer or the key parameters determining the specific relevance of distinct hallmarks in the systems context, and the systems-biologic prerequisites how to specifically unlock the tumor-promoting phenotypes. Therefore, it is only possible to draw on an effect-based description of anakoinosis, that records quantitative and qualitative changes in tumor phenotypes, here inflammation control, differentiation induction, and clinical outcome parameters, such as long-term disease control, CR and cCR in metastatic r/r neoplasias (28).

For the assessment of drug interactions in tumor tissue editing schedules, it is decisive that the single drugs have no or limited monoactivity, that CR or cCR induction with single components of the editing schedules may be excluded in r/r neoplasias, and that the tissue targets are communicatively linked tumor networks whose network characteristics must be readjusted at multiple heterogeneous localizations in case of metastatic r/r neoplasias. Thus, each therapeutic element, metronomic low-dose chemotherapy/azacitidine, and dual/triple transcriptional modulation pro-anakoinotically contributes to reprogram hallmarks of cancer for induction of tumor cell death, and for achieving M-CRAC control by re-integration of on-topic edited hallmarks in the tumor systems context (**Table 3**) (11, 40, 64–67).

### Side effects of pioglitazone and the advantage of low dose application

General side effects of pioglitazone are weight gain and fluid retention (2). Therefore, creatinine at inclusion had to be <132.6 µmol/L and serum albumin >25g/L (39). Only in rare cases pioglitazone had to be discontinued due to fluid retention (68). Hypoglycemia did not occur in normoglycemic patients. In case of patients with diabetes mellitus, the additional anti-diabetic medication was successfully adapted. Elevation of serum creatinine occurred if pioglitazone was combined with a cyclooxygenease-2 inhibitor (2, 69).

Scheduled dose reductions were intended for each drug within the respective editing schedules. If clinically necessary, the COX-2 inhibitor was discontinued after preceding scheduled dose reduction. Also, patients with reduction of pioglitazone to 15 mg daily during treatment achieved CR in r/r neoplasia. Therefore, the initial dose of 60mg pioglitazone in early editing trials was reduced to 45mg as starting dose (39, 70). The minimal active dose of pioglitazone could not be evaluated. Fluid retention during additional dexamethasone treatment caused scheduled dose reduction of dexamethasone in the first step. Discontinuation of pioglitazone treatment due to pioglitazone related side effects led to study termination in rare cases (68). Urothelial carcinoma was not observed during pioglitazone treatment and follow-up (**Table 1**).

## Pre-clinical and clinical data on the contribution of pioglitazone to M-CRAC control

All discussed contributions on PPARα/γ activation with pioglitazone are to be considered conditionally, even if interactions with other cytokines or nuclear transcription factors have been studied experimentally (59). All these dual or triple transcriptional modulation therapies alone, would not induce CR or cCR in r/r neoplasias. However, induction of tumor stress response with low dose chemotherapy in addition to tumor specific transcriptional modulation facilitates control of r/r neoplasias (**Figure 1**).

M-CRAC re-establishes tumor promoting hallmarks of cancer, particularly, following maximized apoptosis induction with pulsed tumor therapies. Tumor cell death inducing therapies promote inflammation, hypoxia, ROS production and may additionally activate the Phoenix rising - caspase-3- cytosolic phospholipase A (2) alpha (cPLA-2)-COX-2-PGE-2-STAT3 pathway to reestablish compensatory tumor regrowth by establishing all disease traits described with M-CRAC (12, 71–73).

As countermeasure, therapeutic PPARα/γ activation reprograms in context with the concerted activity profile of tumor tissue editing approaches hallmarks of cancer, represses important transcription factors, like STAT3, NF-kB, AP-1, PI3K/Akt, HIF1α and NFAT and decreases the expression of TNF-α, TGF-β, IL-6, IL-8, VEGF, iNOS, as indicated by preclinical data (40, 74–76).

PPAR α and γ are positioned at crossing points between lipid metabolism and transcription, balancing and reciprocally cross-linking developmental homeostatic processes, that are established between classic cancer hallmarks, e.g., between tumor cell differentiation and immune surveillance, or immune response and the inflammatory status (26, 77–79). The expression profiles of PPARs in tumor tissues under changing phenotypic conditions are not well studied, yet (56).

M-CRAC control with pioglitazone was feasible independently of the chosen editing procedure, that was either directed at inflammation control or differentiation induction. Responses to edited inflammation control comprised cCR in metastatic r/r RCCC, mLCH and HL and long-term tumor control, even without objective response, e.g., in uveal melanoma (21–23, 30, 80). Therefore, M-CRAC control with pioglitazone provides a unique treatment quality of tumor tissue editing schedules.

### Pioglitazone and inhibition of metastatic growth

Pioglitazone, both the PPARα and PPARγ agonistic component, inhibit carcinogenesis, tumor progression, proliferative capacity of metastasis-initiating stem cells, migration, invasion and remodel the extracellular matrix and angiogenesis (3, 77, 81). Attenuation of the Wnt/β-Catenin signaling, and reduction of the non-canonical NF-κB activity contribute to the M-CRAC inhibiting profile (43, 82–86) (**Figure 1**).

Clinically, pioglitazone inhibits colony-formation of stem cells in chronic myelocytic leukemia and may induce differentiation via CD44, an epitope, frequently expressed on cancer stem cells (63). Metabolic dependencies between tumor cells and the adjacent microenvironment promote heterogeneous metabolic phenotypes during development of therapy resistance and metastases. Metabolic reprogramming with glitazones might lower in experimental models the efficacy of the metastatic process (87–90).

The addition of pioglitazone in tumor tissue editing schedules may control metastatic spread in histologically different r/r tumor treated. In >60% of patients with tumor progression following tumor tissue editing, progression took place at the original tumor sites (21).

### Pioglitazone and inhibition of sustained proliferation, cell death and differentiation induction

#### Growth attenuation

Restoring the expression of tumor suppressors, such as PTEN with PPARγ agonists attenuates tumor repopulation, prevents residual tumor cells and aggressivity of tumor cells (86, 91) (**Figure 1**). Simultaneously, PPARγ agonists reduce the activity of PI3K/Akt pathway and down-regulate Bcl-2 (92).

Less recognized is the potent immunoregulatory role of PPARγ regarding all immune cells that contributes to improved immune surveillance and growth attenuation (93–96) (**Figure 1**). PPARγ agonists are decisively shaping the molecular phenotype of the whole T-cell repertoire, of macrophages, and dendritic cells inclusively their communicative behavior (97, 98) and favor the M2 phenotype of macrophages which is associated with the expression of TGF-β, that is involved in M-CRAC promotion (99, 100). In all subtypes of T cells and macrophages, PPARγ agonists regulate the expression of genes involved in lipid metabolism and transport, e.g., the class B scavenger receptor CD36, besides FABP4, LXRA, and PGAR (101–103). Differentiation induction in non-PML AML by including pioglitazone could alter the antigenicity of leukemia cells (30, 34).

Enhanced PPARγ-mediated lipid antigen presentation facilitates the activation of iNKT cells (76, 104–107). Promoted by PPARγ agonists, fatty acid up-take and oxygenation may derepress effector T-cells and favor immunologic tumor response (108, 108).

#### Cell death

PPARα and PPARγ belong to ferroptosis related genes (109). Clinical trials on tumor editing, including PPARalpha/gamma agonists have shown that apoptosis resistance may be overcome by reprogramming cancer hallmarks. Apoptosis may be bypassed via differentiation induction (30). Pro-apoptotic effects of PPARγ ligands have been proven by multiple pre-clinical studies (110). PPARγ activation decreases the expression of cyclin D1, thereby stopping the cytosolic β-catenin accumulation and may induce G2/M cell cycle arrest (111).

#### Differentiation

Pioglitazone alone may not induce clinically relevant differentiation (84). Nevertheless, pioglitazone contributes to balance proliferation and differentiation. Highly differential pathways may be involved in differentiation induction dependent on tumor histology, favoring the use of combined biomodulatory therapies including pioglitazone (**Figure 1**) (30, 112, 113).

#### Pioglitazone and prevention of drug resistance

Tumor cell death, and hypoxia following apoptosis inducing therapies contribute to a phenotype favoring drug resistance (114–116). In the first step phenotypic alterations of tumor and stroma cells arise on epigenetic basis (117, 118). PPARγ activation may normalize epigenetic and transcriptional regulation related to altered lipid metabolism (119, 120). The development of genetic resistance is based on genetic instability of tumor cells.

By attenuating the detrimental effects of pro-inflammatory cytokines, pioglitazone contributes to avoid the development of resistance in a concerted approach with other biomodulators (121). Further, therapeutic attenuation of Wnt signaling may be an important approach for resolving resistance (122).

In a clinical trial on r/r multiple myeloma the addition of pioglitazone in IMiD resistant disease resolved IMiD resistance while continuing IMiD therapy combined with pioglitazone (123). Experimentally and clinically studied is the method to overcome imatinib resistance in CML, defined as no achievement of MRD negativity. By targeting CML stem cells and the STAT system, pioglitazone even allows the discontinuation of the combination therapy pioglitazone, imatinib (124, 125).

The unimpeded passage of pioglitazone through the blood-brain barrier must be suggested, as cCR may be achieved in multisystem Langerhans cell histiocytosis (mLCH) and cerebral involvement (23, 126).

#### Addressing heterogeneity of tumor cell niches

Huge timely and spatially diversified activity profiles of pioglitazone in heterogeneously constituted tumor cell niches might be expected when considering the context-dependent activity profiles of pioglitazone, as indicated by pre-clinical data (26, 127, 128). In contrast, concertedly targeting cancer-associated hallmarks by reprogramming techniques including pioglitazone may induce cCR, even in r/r HL, although all HL patients received prior local irradiation (21–23, 80). Control of obstacles given by molecular-genetic/genetic tumor cell and stroma heterogeneity in metastatic tumor disease, is facilitated by the concerted action of metronomic low-dose chemotherapy that provides important prerequisites for the activity profile of dual/triple transcriptional modulation with pioglitazone (21–23, 44).

## The contribution of pioglitazone to long-term tumor control, CR or cCR by resolving tumor-promoting inflammation or inducing differentiation

Metronomic low dose chemotherapy plus pioglitazone alone or in combination with additional transcriptional modulators, i.e., dexamethasone, ATRA, or interferon-α contributes to therapeutically unlocking the tumor tissues’ phenotype via anakoinosis, causing CR and cCR and long-term tumor control in r/r neoplasias by attenuating tumor-promoting inflammation, differentiation induction, M-CRAC control, and non-oncogene addiction in edited tumor tissue (**Tables 1**–**3**; **Figure 1**) (21–23, 25–28). Trials on tumor tissue editing were performed as indicated in **Table 1** (21–23, 30, 35, 38, 39, 68, 70, 80, 123, 129–134).

### Control of systemic tumor-promoting inflammation by tumor tissue editing

Control of tumor-promoting inflammation could be achieved with histology adapted and individually designed editing protocols in four histologically quite different r/r metastatic neoplasias, melanoma, RCCC, hepatocellular carcinoma, mLCH and HL (**Table 3**) (22, 23, 37, 38).

### Melanoma and inflammation control

In a randomized comparison for r/r metastatic melanoma, the addition of a COX-2 inhibitor for inflammation control to metronomic low-dose chemotherapy emerged significantly inferior compared to intensified anti-inflammatory therapy with COX-2 inhibitor plus pioglitazone concerning PFS (**Table 3**) (38, 135). OS in r/r metastatic melanoma was significantly correlated with CRP serum response (38, 136). In a preceding trial including pioglitazone, one cCR was reported in r/r melanoma (137). COX2/PPARγ tissue immunoreactivity significantly increases stage-dependently from primary melanoma to metastases. Strong PPARγ immunoreactivity in melanoma cells was associated with improved PFS in retrospective analysis (50). Further, it could be shown that improvement of ECOG status and cachexia control in melanoma patients may be mediated by pioglitazone/COX-2-related disruption of platelet derived aberrant serum protein and lipid crosstalk between lipolysis of fat tissue and muscle wasting associated oxidative stress, that are both mediating cachexia (138, 139).

### Intensified inflammation control in RCCC

Intensification of anti-inflammatory therapy with low-dose interferon-α in addition to pioglitazone in a trial on r/r metastatic RCCC led to early CRP serum response in objective responders, also in patients with delayed CR (**Tables 2**, **3**) (21, 35, 37, 140–144). In contrast to stable disease (SD) as best response in the preceding trial without low-dose interferon-α, cCRs were achieved with intensified inflammation control (35, 37, 145). Interferon-α, prednisone and 5-FU are only moderately active in RCCC (142).

### mLCH, an inflammation-triggered neoplasia

A strong inflammation-triggered tumor promotion via activation of the nuclear factor kappa B (NFκB) pathway is well known in mLCH (146, 147). Pioglitazone plus dexamethasone induced early CRP response in some patients followed by cCR, even in case of cerebral involvement (21, 23).

### Editing inflammation control and non-oncogene addiction in r/r HL and melanoma

The editing schedule for Hodgkin’s lymphomas was identical to the schedule used in r/r mLCH but supplemented by an mTOR inhibitor (**Table 2**). Response to editing therapy was closely related to serum CRP response (148–150). In contrast to mLCH, only the addition of everolimus to the mLCH schedule induced cCR in r/r HL (22, 23, 151, 152). Importantly, mTOR inhibition has been reported to be inefficacious in addition to pulsed chemotherapy (**Table 2**) (22, 151, 153). Editing non-oncogene addiction for successful clinical access of mTOR inhibition might also enhance immunosurveillance in r/r HL (154).

By adding temsirolimus to the pioglitazone arm of the melanoma editing schedule, long-term melanoma control was achieved in patients with extensive liver metastases of uveal melanoma by efficacious M-CRAC control (79, 80, 155).

### Leukemia-specific control of sustained proliferative signaling by differentiation induction

Differentiation induction in leukemias with driver mutation is suggested to be locked for therapeutic reprogramming. The prototype for successful differentiation induction is the PML. PML may be controlled by the classic drug, ATRA. In case of ATRA therapy for PML, only the combined use with an additional drug, e.g., arsenic trioxide, may induce cCR (156). In another disease with a typical driver mutation, chronic myelocytic leukemia (CML), pioglitazone combined with imatinib may overcome minimal residual disease in patients not achieving molecular CR (124).

Differentiation induction in non-PML AML without typical driver mutation Also non-PML AMLs without actionable mutation are accessible for differentiation induction. R/r non-PML AML patients may achieve molecular-genetic or hematologic remission with azacitidine, ATRA and pioglitazone (**Tables 2**, **3**) (30, 34). In the experimental setting, only the addition of pioglitazone to the combined editing approach facilitates differentiation and regain of phagocytic activity of differentiated, neutrophil-like blasts *in vitro* (36).

Moreover, blasts differentiated to neutrophil-like cells regain *in vivo* phagocytic activity and may resolve during study treatment prior to study medication acquired pneumonia. The clinical observation impressively demonstrates the generally low-toxicity range of tissue editing approaches and the regain of functionality by differentiation induction in AML blasts (30, 34).

Differentiation induction with pioglitazone in tumors In an animal model on breast cancer, it could be shown that the combination of pioglitazone with a MEK inhibitor may induce tumor differentiation, transdifferentiation of tumor cells to adipocytes by epithelial-mesenchymal transission (42, 157).

Thus, editing techniques including pioglitazone may induce specific types of differentiation in quite different neoplasias. However, editing schedules must be specifically adapted to tumor histology. Although, pioglitazone is discussed as apoptosis or ferroptosis inducer, differentiation inducing editing schedules may bypass common cell death pathways via differentiation (30) **Figure 1**.

## Tumor-type agnostic pro-anakoinotic access to network-based ligand-receptor cross-talks via pioglitazone

Due to treatment failure to an inflammation suppressing editing schedule including pioglitazone, an individual therapeutic adaption was successfully performed in refractory mLCH by the addition of low-dose interferon-α to pioglitazone and discontinuation of dexamethasone (**Tables 2**, **3**) (22). The adaption was considered due to the observed strong inflammation control of interferon-α and pioglitazone, administered in a RCCC editing schedule. In the mLCH schedule, only the cytotoxic drug of metronomic chemotherapy was substituted by a continuously administered alkylating agent, trofosfamide, instead of capecitabine, as used in the RCCC trial. Adaption of the editing schedule led to cCR, here, in a little child with severe multisystem involvement of refractory LCH, including LCH mediated severe bone marrow and liver failure, disease traits, which were primarily intended for combined liver and bone marrow transplantation (22, 37).

Histology-related accessibility of editing approaches controlling inflammation or inducing differentiation and obviously shared phenotype-maintaining ligand-receptor cross-talks among histologically quite different tumors show that therapeutic editing including pioglitazone for reprogramming tumor hallmarks is tumor-type agnostic, independently of the editing strategy, inflammation control or differentiation induction (**Table 2**).

## Low-dose metronomic chemotherapy prerequisite for successful dual/triple transcriptional editing including pioglitazone

CR or cCR induction with dual/triple transcriptional modulation in histologically quite different r/r neoplasias without driver mutation was only possible in combination with metronomic low-dose chemotherapy (11, 63, 156).

The possibility for unlocking tumor phenotypes via the tumor-type agnostic activity of pioglitazone with differential recombinations of additional transcriptional modulators underlines the novel unique activity profile of low-dose metronomic chemotherapy, namely, providing pleiotropic cancer hallmark-related tumor tissue responses, i.e., altered cytokine and transcriptional repertoires in tumor tissues, including altered PPARγ expression, as prerequisite for specified tumor tissue editing with ligands of nuclear transcription factors or cytokine receptors (21–23, 25, 37, 56, 158–160) (**Table 3**). The fact that induction of cCR was possible also in cases with scheduled dose reductions up to > 66% of the metronomic starting dose, irrespectively of the used cytotoxic drug, trofosfamide, treosulfan or capecitabine, underlines that apoptosis induction could not be the primary purpose of low-dose metronomic chemotherapy (**Table 2**) (21–23, 37). Importantly, at the end of a 3 or 4-week cycle the trials did not achieve the cumulative dose of corresponding pulsed chemotherapy schedules, and scheduled dose reductions were frequently performed, in some patients primarily, due to multiple preceding therapies (**Tables 2**, **3**) (21–23, 30, 38, 44, 56, 80, 161, 162).

Drawing on observations in biology, the clinical data on low-dose metronomic chemotherapy suggest that pleiotropic stress responses to metronomic chemotherapy limit tumor tissue plasticity, probably decrease functional heterogeneity of tumor cell niches, as tissue stress generally induces a tighter phenotype. Metronomic chemotherapy could promote via stress response phenotypic integration of inflammation or differentiation within editing schedules, and consecutively may serve as an enhancer of pro-anakoinotic effects induced by combined treatment with nuclear receptor agonists or cytokines (**Figure 1**) (26, 162–164).

In diseases with driver mutation, such as chronic myelocytic leukemia (CML) the addition of pioglitazone to imatinib is sufficient to eliminate minimal residual disease (63, 124). Metronomic chemotherapy is no prerequisite for the biomodulatory activity of pioglitazone in CML.

Whether a MEK inhibitor plus pioglitazone, probably in combination with metronomic chemotherapy, is sufficient to control metastatic breast cancer in humans via differentiation induction must be further evaluated (42).

## Discussion

Metronomic low-dose chemotherapy or epigenetic modeling with azacitidine combined with differential recombinations of pioglitazone with transcriptional modulators initiate tumor-specific reprogramming of cancer hallmarks, i.e., anakoinosis, here exemplified by tumor-associated inflammation control or differentiation induction. Thus, tissue editing techniques disclose that the wide-ranging functions of PPARα/γ agonists in tumor tissues may be selectively focused on differential reprogramming patterns of cancer hallmarks, induction of tumor cell death, and on facilitating edited non-oncogene addiction. Now, pioglitazone reaches clinical relevance in oncology if combined with appropriate additional biomodulators, even in relapsed or refractory neoplasias (**Figure 1**).

Therapeutic control of M-CRAC in r/r neoplasias affords tumor tissue editing with metronomic low dose chemotherapy and histology-adapted dual or triple transcriptional modulation, particularly in case of r/r tumors without driver mutation and complex genetic aberrations. By selecting histology-specific editing protocols tumor phenotypes may be specifically unlocked with respective dual/triple transcriptional modulation. Clinically, main therapeutic emphasis may be, as shown, either differentiation induction, enhancement of immunosurveillance, metabolic reprogramming or inflammation control.

Thus, editing procedures repurpose the function of metronomic low-dose chemotherapy and add an important, yet less considered activity profile of metronomic chemotherapy, induction of stress response in tumor tissues as prerequisite for combined transcriptional editing (**Figure 1**) (56).

Editing techniques reveal that tumor-specific networks of ligand-receptor cross-talks for maintaining tumor phenotypes provide unique tumor systems characteristics. Tumor phenotype-maintaining cross-talks facilitate concerted ‘targeted’ pro-anakoinotic tumor systems access. Anakoinosis may be initiated by induction of tumor stress response combined with dual/triple transcriptional modulation including pioglitazone.

Drugability of tumor phenotypes for therapeutically exploiting tumor plasticity is in line with experimental data derived from Zebrafish models showing that e.g., the re-establishment of embryonic microenvironment may determine tumor cell fate (165).

Editing of cancer hallmarks with pioglitazone may directly induce tumor cell death or alternatively, provides novel access for tumor cell death induction via edited non-oncogene addiction (21–23, 38, 80). However, editing may also facilitate control of metastasis, post-therapy cancer repopulation and acquired cell-resistance and genetic/molecular-genetic tumor cell heterogeneity (M-CRAC) (11). That means tumor promoting hallmarks remain long-term silenced, as shown in uveal melanoma (80). These differential response patterns reveal that on-topic edited hallmarks of cancer are both, differentially constituted and interconnected within the pattern of cancer hallmarks, therefore, specifically accessible with recombinations of transcriptional modulators. The differential activity profiles of pioglitazone in on-topic editing approaches highlights a further individual tumor characteristic, namely, the specific communicative integration of distinct cancer hallmarks within the whole pattern of hallmarks (22, 23, 30, 38, 163).

The unlocking technique of tumor phenotypes with pioglitazone plus/minus additional transcriptional modulators must be currently selected according to tumor histology. However, ligand-receptor cross-talks, maintaining cancer hallmarks, may be shared among quite different tumor histologies revealing a tumor-type agnostic therapeutic access of pioglitazone across different histologic tumor types, e.g., melanoma, renal clear cell carcinoma, Langerhans cell histiocytosis, Hodgkin’s lymphoma and acute myelocytic leukemia (166).

Tissue editing approaches, and therapies including maximum tolerable doses to induce maximized apoptosis induction, seem to be mutually exclusive, as exemplified from the literature for mTOR inhibitors in r/rHL, interferon-α in RCCC or epigenetic modifiers (21, 22, 30, 37, 118, 153, 167–169).

Metronomic low-dose chemotherapy provides prerequisites for the therapeutic dual/triple transcriptional modulation to unfold combined transcriptional systems activity in tumors. The configuration of cancer hallmarks, their ‘integration quality’, i.e., how hallmarks of cancer are communicatively cross-linked, and their particular importance within the tumor systems context specify outcome of tumor tissue editing and the context-dependent therapeutic use of pioglitazone. Monitoring these tumor-promoting hallmark qualities may specify tumor tissue editing and also the context-dependent therapeutic use of pioglitazone (22, 23, 30, 38, 163).

Basic research is now challenged to provide data on network-based ligand-receptor cross-talks in tumor tissue compartments for specifying editing schedules according to the individual configuration and integration of hallmarks, to evaluate the most suitable pro-anakoinotically druggable hallmarks and to exploit the most powerful activity profiles for pioglitazone to finally efficaciously reprogram the tumor tissue’s network-based transcriptional ‘Achilles heel’ (44, 163, 170).

Experimental data derived from tumor tissues show that hallmarks of cancer are in homeostatic balance by spatially organized, multicellular inflammatory and immune modulatory hubs and that cellular heterogeneity in tumor tissues is not at random (171, 172). These hubs might serve as organized access for tumor tissue editing schedules, also for pioglitazone as immune modulator, inflammation inhibitor, metabolic regulator and differentiation inducer (172). Particularly, proteomic platforms may enable to test differentiation induction or inflammation control and to uncover ways for edited non-oncogene addiction in a highly personalized manner (20, 29, 173).

Integration of pioglitazone in tissue editing approaches marks the starting point for concertedly targeting the communicative context of multiple tumor cell compartments and their tumor-typical networks of ligand-receptor cross-talks for systematically controlling metastatic r/r neoplasias up to cCR. The pro-anakoinotic active magic bullets including pioglitazone unlock the tumor phenotype, thereby addressing urgent therapeutic problems, such as functional, topographic, and genetic heterogeneity of tumor and stroma cells, tumor cell dormancy, dynamically changing phenotypic competences of tumor cell niches, and M-CRAC (24, 36, 44, 127, 174–176). Alongside, tumor tissue editing with pioglitazone may induce tumor cell differentiation. As differentiated blasts gain phagocytic competence, a broad variety of cell death pathways might be induced in those cells, similar to the repertoire of cell death pathways in granulocytes, including necroptosis and pyroptosis with disintegration of the cellular membrane and non-lytic apoptosis or NETosis (24, 36, 44, 127, 174–177).

The therapeutic paradigm change, namely, evolving tumor phenotypes with tumor-type agonistic tumor tissue editing approaches including pioglitazone in therapeutically stressed tumor tissue, provides a novel expedient technique for controlling r/r metastatic tumors of quite different histologic origin (178).

## Author contributions

DH: Conceptualization, Writing – original draft, Writing – review & editing. FL: Conceptualization, Writing – original draft, Writing – review & editing. TP: Conceptualization, Writing – review & editing. LG: Conceptualization, Writing – review & editing. CG: Conceptualization, Writing – review & editing. AR: Conceptualization, Writing – original draft, Writing – review & editing. DCH: Conceptualization, Writing – original draft, Writing – review & editing.
